# The Early and the Late Operations for Appendicitis1Read before the Lake Erie Medical Society, June 29, 1900.

**Published:** 1900-08

**Authors:** Eugene A. Smith

**Affiliations:** Buffalo, N. Y., 1018 Main Street


### THE EARLY AND THE LATE OPERATIONS FOR APPENDICITIS.[1]

By EUGENE A. SMITH, M. D., Buffalo, N. Y.

APPENDICITIS in the last several years has been in print so often before the public as well as before the medical profession, discussed by both laymen and professional men, that an apology is hereby tendered for presenting so hackneyed a subject before this association. While all agree on the insidious nature of the disease in its development, the violence of its onset and its gravity in the more serious forms, there are many views as to its pathology and prognosis, and there are two views as to treatment which may be classed as the operative and the nonoperative or medical. It has numerous forms or types, its progress is uncertain, its complications are many and its terminations varied. It is not surprising, therefore, that our knowledge of this subject is not yet crystallised into definite rules for our guidance in treatment, based on a definite pathology and consequent prognosis. Daily, however, our statistics increase and from the voluminous writings on the subject we can make more certain conclusions, and hold out to the victim of this disease better and better prospects of recovery along well-defined lines of treatment. That the majority of medical men lean strongly to the operative treatment of appendicitis is not to be denied. Believing in an infection in the appendix as the exciting cause of the diseased processes which ensue, be the predisposing cause any one of several, such as traumatism, foreign body, fecal concrement, bends or twists of the appendix, deficient blood supply, gastroenteritis, colitis and salpingitis and ovaritis extending to the appendix, it is a fact that operative removal of the focus of trouble

---

1 Read before the Lake Erie Medical Society, June 29, 1900.

is the essential element in treatment. This paper can not appeal to men who believe in a vague inflammatory condition in the right lower quadrant of the abdomen, known as perityphlitis or paratyphlitis. They must first become schooled in the pathological teaching that shows the reasonableness of infection and inflammation in the useless blind pouch, rich in lymphoid tissue and prone to inflame, known as the appendix vermiformis. The same teaching points to the rarity of cecal traumatism and ulcer as a cause for retrocecal cellulitis and pus formation, which pus when gathered gives the name perityphlitic or retrocecal, or pelvic, or perinephritic abscess, variously so-called by the older writers.

We may now subdivide the class of men who believe in the operative treatment of appendicitis into subclasses, the two principal ones comprising those who operate early and prescribe medically afterward; second, those who treat by internal medication first, and operate late if they operate at all, being compelled so to do by developments that are only to be treated surgically, such as pus formation or obstruction of the bowel. A subdivision of this latter class comprises those who treat the patient during an attack of appendicitis expectantly with a view to operate in the interval after subsidence of inflammation, before a new attack develops. Other subdivisions could be mentioned. Some men say they never operate, never finding it necessary to do so. They are untruthful, or they are self-deceived and are the most unsafe men to whom patients suffering with this disease can entrust themselves. Another class to be mentioned is composed of men who send a dose or several doses of morphine to patients having colic with cramps and vomiting on the diagnosis made by some friend or relative of the patient, and, who, upon visiting the patient a day or two later occasionally find a patient dying with peritonitis following perforative or gangrenous appendicitis.

Beginning with June 1, 1899, and ending with June 1, 1900, I have seen forty-six cases of appendicitis in consultation with the attending physicians, thirty in number. Some of these men have a large practice and nine of them have called on me for surgical help on two occasions in such cases; two of them calling me three times. Of these cases, ten were not operated upon; in nine cases, because the attack was mild and the patients chose to await the subsidence of inflammation for an interval operation. It is of interest to note that only one of them has so far come for operation, and in this case it was because a new attack developed. In the tenth case the father of the patient declined operation for his son, a boy of 17, because another son had died of this disease after operation. This boy also died after two weeks of suffering, the father stubbornly refusing operative help. Beyond doubt some of the medical men who called me for severe cases, have carried milder cases through without consultation or operation, and some may have called other surgeons in some of their cases, or may have failed to diagnosticate some mild cases of appendicitis under their observation. It can not therefore be said that these cases represent all the experience in appendicitis of thirty physicians for one year, but it can be said that these cases represent 90 per cent. of the cases that twenty of these men have had in one year. Again, it must be allowed that not all of the thirty-six cases operated upon would have died without operation. Some of the early cases operated upon would have been successfully treated by morphine or by cathartics beyond a doubt, as far as subsidence of acute inflammation in the attack under consideration is concerned. I venture to assert, however, that no one of them would take back his appendix for a chance to be where he was before its removal. Of those who died, no one, so far as surgical judgment can determine, would have recovered without operation.

| Month. | No operation. | Operation. | | | Drainage for Pus. | Result. | Complications and Sequelæ all in Late Cases. |
|---|---|---|---|---|---|---|---|
| | | Interval Cases. | Early Cases. | Late Cases. | | | |
| 1899. | | | | | | | |
| June | 1 | ...... | ...... | 1 | 1 | 1 death (non-op. case). Recovery. | Fecal fistula, since healed. |
| July | ...... | ...... | ...... | 1 | 1 | " | Fecal fistula, since healed. |
| August | ...... | ...... | ...... | 1 | 1 | " | Hernia (one). |
| September | 2 | ...... | 1 | ...... | 1 early. | " | |
| October | ...... | 1 | 1 | 1 | 1 late. | " | |
| November | 2 | ...... | ...... | 3 | 3 | " | Cystitis. Broncho-pneumonia. Hernia. |
| December | 1 | ...... | ...... | 2 | 2 | 1 death. | |
| 1900. | | | | | | | |
| January | ...... | ...... | ...... | 1 | 1 | Recovery. | Hernia. |
| February | 3 | ...... | ...... | 2 | 2 | 2 deaths. | |
| March | ...... | ...... | 1 | 5 | 5 late. | Recovery. | Cystitis. Broncho-pneumonia, 2 cases. |
| April | ...... | 1 | 1 | 4 | 4 late. 1 interval. | 1 death (late). | Fecal fistula (one not yet healed). |
| May | 1 | 1 | 3 | 5 | 5 late. | Recovery. | Broncho-pneumonia and nephritis. |
| | 10 | 3 | 7 | 26 | 28 | | |

All late cases needed drainage. One death without operation. Four deaths after operation, all late cases.

By reference to the table here printed and upon analysing these cases there is a striking difference to be seen in the steps of the operative treatment, in the complications, in the sequelæ and in the prognosis between cases operated upon early or in the first seventy-two hours, and those operated upon later than the third day or after seventy-two hours. It is of course an arbitrary division as to time, when we call cases operated upon before the fourth day early cases, but it fairly serves a purpose as regards prognosis for the majority of cases. In many slowly developing subacute cases an early operation is possible at the end of a week or even two weeks. On the other hand, fulminating perforative or gangrenous cases with spreading general peritonitis are late cases at the end of twenty-four hours from their first clinical manifestations.

Many operators use the term late-early cases for patients in the second and third day of the disease, depending on the severity of the case, and early-late cases for those in the fourth, fifth, sixth or seventh day, again depending on the nature of the case; here especially depending on the amount and the location of pus. In explanation of these distinctions it may be said a fulminating case is already late from the operative view-point of good prognosis if the disease is two days old, yet it is an early case according to time. So again an acute case with adhesions walling in a small pocket of pus is a late-early case for a safe operation on the fouth day, becoming more and more a late case as the abscess enlarges. It is in this class of cases that operators are now warmly arguing, as to the advisability of searching for the appendix and removing it, making the so-called complete operation, or of evacuating pus without removing the appendix, making in many cases a far easier and safer, but incomplete operation.

In this latter class it is later necessary to remove the appendix or to leave the patient in danger of another attack of appendicitis. This has occurred in two of the series of cases submitted in this paper. One was a boy of five years and three months old, from whom I removed a perforated appendix on the second day of a second attack, a large pelvic abscess having been evacuated by Dr. Mynter about one year before, when the boy was four years and four months old. In the second case the patient was a boy of nine years and had a relapse of appendicitis, requiring an appendectomy two weeks after evacuation of a large pelvic abscess, due to his first attack of appendicitis.

It is a fact that obliteration of the appendix may occur through repeated attacks of inflammation, but this necessarily occurs in only a small proportion of cases, so small in fact that it can not be counted in considering prognosis. Again, a very few cases of first attacks of appendicitis are not followed by later attacks, but again the proportion of such cases is too uncertain to apply to a case in hand to be considered in prognosis as a safe outcome for the patient.

When abscess forms after appendicitis and perforation due to the sloughing out of a fecal concrement or foreign body occurs it is possible for spontaneous cure to follow if the pus breaks into bowel, bladder or vagina, or onto the surface in the groin, or, if retrocecal, in the loin. Second attacks may occur in this class of cases, except in a small proportion in which walling by adhesions of proximal good appendix occurs and sloughing and exfoliation of the portion distal to a concrement or stricture occurs. In this class of cases occur a large number of fecal fistulæ more or less slow to close, dangerous, extremely disagreeable, and eminently unsatisfactory to treat.

When we now turn to the question of operative work for appendicitis, what are the reasons for and against early operation? In favor are results, which in good hands are said to be over 90 per cent. of recoveries, if done early or in the interval. In ten interval cases, I have had no deaths. In one of the three cases, in the table of cases here reported, the patient had had several severe attacks and some less severe ones, making thirteen attacks in less than a year. He came from Dundas, Canada, no positive diagnosis having been made, with a mass in the right iliac fossa, from which I removed a broken down appendix. I judged it best to drain because of the excessive amount of inflammatory new formation. His was the only interval case I have ever drained. Reference to the table shows no mortality in seven early cases before pus formation in which no drainage was used.

Dread of operation and danger of anesthetic being overcome and operation completed, moderate pain, occasional hiccoughing, some troublesome constipation, moderate fever, occasional stitch or wound suppuration, a brief afterperiod of colicy pain due to adhesions and vague dyspeptic conditions for a few weeks or months sum up the post-operative discomforts. In no one of these two classes of interval and early cases has rupture in the line of incision thus far occurred.

Against operation may be mentioned the danger due to it, post-operative suffering, hernia, and the mortality already given, which is less than 10 per cent. according to the most unfavorable figures. That there is no danger in operation I do not believe. An occasional case with adhesions, or pocketed pus due to one or more attacks of appendicitis, may cause fatal complications from shock, hemorrhage, sepsis or obstruction of the bowel. When, however, we compare this mortality with that of unoperated cases, we find the most favorable statistics pointing to 20 to 40 per cent. of deaths in 100 cases, granting that 60 to 80 recover from a first attack. As regards suffering it is enough to say it does not compare with that endured by patients not operated upon. Unoperated cases must be more or less constantly and completely narcotised with morphine, which drug may be judiciously used in far smaller quantities in operated cases to control suffering with far better satisfaction to patient and attendant. As regards hernia, no one will claim any comparison between early and late cases in this respect. Late operation with drainage in a large proportion results in formation of hernia in the longer line of cicatricial tissue, at least partly closed by granulating tissue in the healing process. In a clean incision, neatly approximated in early cases, hernia should be and is extremely rare.

If we now consider appendicitis from the standpoint of the men who delay, we enter upon a wide field of the complications occurring with and following appendicitis.

To begin with, late operators lose all of the fulminating cases of gangrenous and perforative appendicitis, and those with general peritonitis, or they may save a small proportion of them by precarious work in the nick of time by a late-early operation. Possibly 60 to 80 per cent. of first attacks subside without pus formation, but out of this number 30 to 50 per cent. will have a second attack, and in due time of the remainder an unknown percentage will have a third and then a fourth attack and each time a small percentage will have a dangerous form and require operation or they will die. It is needless to say operators recognise that with each new attack, complications from adhesions are more frequent and liability to sepsis and danger of bowel obstruction are more pronounced.

In the 20 to 40 per cent. of cases of first attack, in which sepsis plays a part to the extent of pus formation, the delayed operation is sure to be done through a longer incision than in early operation, owing to the adhesions which form. Healing in these cases is prolonged from two weeks in early operation to three, six or eight weeks in late operation, and, moreover, patients are emaciated, anemic and weak for weeks and months afterward.

With pus formation, metal tube or gauze drainage must be employed. For these reasons hernia in the line of the incision is often seen. In the twenty-two cases operated upon and drained for large pus collections, or drained after washing out the peritoneal cavity and pelvis for sero-purulent forms of peritonitis with no limiting adhesions, I now have three cases of hernia for the relief of which my patients are obliged to wear a bandage of the ventral hernia truss pattern. It is still too early to say that several more in this series may not develop hernia.

Infection elsewhere and pus collections due to metastasis or to spread of inflammation by continuity or contiguity are likely to occur in delayed cases. One case had retrocecal abscess two weeks following a pelvic abscess present when the perforated appendix was removed and a fecal concrement found. Fully three weeks later another abscess, due to spread of sepsis in cellular tissue was found and opened above the liver and below the diaphragm, and here a second fecal concrement, plainly from the appendix, removed seven weeks before, was washed out in the pus. Following one septic case, in a young girl, a large parotid gland abscess developed in the second week and was opened, the patient thereafter doing well excepting that she made a slow recovery from a facial paralysis owing to involvement of the facial nerve. Other sequelæ of cases coming late for operation of the nature of metastatic infection or of secondary infection owing to lowered vitality, are two cases of broncho-pneumonia, one case of erysipelas, first appearing around the wound and then on the face spreading to the scalp, two of cystitis, probably due to catheterisation, and one of acute nephritis with broncho-pneumonia and pleurisy.

It is in this class of cases, also, that dangerous adhesions producing bowel obstruction are most frequently seen. I lost one such case. This man had a long illness, finally getting over a first attack sufficiently to attend to his legal work, but he died of obstruction of the bowel and peritonitis, after operation for a second attack in which the appendix became gangrenous.

Of fecal fistula, I have records of three cases, all occurring either with or without operation in late cases. One was a case of spontaneous opening of an appendicular abscess just above Poupart's ligament. The other two followed drainage of the iliac fossa after amputation by circular ligature of a gangrenous appendix.

In summing up I find in ten cases of appendicitis not operated upon that one died. In thirty-six cases operated upon, four died. In ten interval and early cases none died. Four died of the twenty-five late cases, one from pylephlebitis, one from obstruction of the bowel and peritonitis, one from general peritonitis which was present at the time of operation, and one from localised pus formation in the infrahepatic and perisplenic tissues with general peritonitis, pus having been free in the peritoneal cavity at the time of the operation.

In conclusion, I urge all medical men to join the class of early operators. Do not wait for the interval. In one hundred cases you will lose a certain number by so doing. Authorities differ on the number, but the figures range from 5 to 40 per cent. of deaths. In this paper I have purposely used percentages in wide ranges, because authorities do not agree and my own statistics are still too small for general conclusions. One man may see case after case of subacute appendicitis recover from the attack without operation, and then see several fulminating cases with an equal percentage of mortality under the same treatment. Surgery fails in some cases, but beyond the possibility of a doubt, the removal of the cause is certainly a safer procedure in appendicitis than is medical treatment with surgery held in the background as a reserve to be called upon to cover the retreat when the battle is lost.

1018 MAIN STREET.

